# Bridging the gap between omics and earth system science to better understand how environmental change impacts marine microbes

**DOI:** 10.1111/gcb.12983

**Published:** 2015-07-28

**Authors:** Thomas Mock, Stuart J. Daines, Richard Geider, Sinead Collins, Metodi Metodiev, Andrew J. Millar, Vincent Moulton, Timothy M. Lenton

**Affiliations:** ^1^ School of Environmental SciencesUniversity of East AngliaNorwich Research ParkNR4 7TJNorwichUK; ^2^ College of Life and Environmental SciencesUniversity of ExeterEX4 4QEExeterUK; ^3^ School of Biological SciencesUniversity of EssexWivenhoe ParkColchesterCO4 3SQUK; ^4^ Ashworth LaboratoriesEdinburgh UniversityEH9 3JFEdinburghUK; ^5^ SynthSys and School of Biological SciencesEdinburgh UniversityEH9 3BFEdinburghUK; ^6^ School of Computing SciencesUniversity of East AngliaNorwich Research ParkNR4 7TJNorwichUK

**Keywords:** evolution, genomics, microbes, modelling, ocean

## Abstract

The advent of genomic‐, transcriptomic‐ and proteomic‐based approaches has revolutionized our ability to describe marine microbial communities, including biogeography, metabolic potential and diversity, mechanisms of adaptation, and phylogeny and evolutionary history. New interdisciplinary approaches are needed to move from this descriptive level to improved quantitative, process‐level understanding of the roles of marine microbes in biogeochemical cycles and of the impact of environmental change on the marine microbial ecosystem. Linking studies at levels from the genome to the organism, to ecological strategies and organism and ecosystem response, requires new modelling approaches. Key to this will be a fundamental shift in modelling scale that represents micro‐organisms from the level of their macromolecular components. This will enable contact with omics data sets and allow acclimation and adaptive response at the phenotype level (i.e. traits) to be simulated as a combination of fitness maximization and evolutionary constraints. This way forward will build on ecological approaches that identify key organism traits and systems biology approaches that integrate traditional physiological measurements with new insights from omics. It will rely on developing an improved understanding of ecophysiology to understand quantitatively environmental controls on microbial growth strategies. It will also incorporate results from experimental evolution studies in the representation of adaptation. The resulting ecosystem‐level models can then evaluate our level of understanding of controls on ecosystem structure and function, highlight major gaps in understanding and help prioritize areas for future research programs. Ultimately, this grand synthesis should improve predictive capability of the ecosystem response to multiple environmental drivers.

## Introduction

The marine ecosystem is a fundamental part of the Earth system, which is both responding to human‐induced global change and affecting its magnitude. As of 2000, land‐use change had added 34 Pg C to the atmosphere, whereas the ocean had absorbed 124 Pg C from anthropogenic emissions (House *et al*., 2002). Absorbing this extra carbon dioxide acidifies the ocean, making it less hospitable for calcifying organisms such as corals, molluscs, echinoderms, fish and calcifying algae (e.g. Kroeker *et al*., 2013). The oceans are also warming and becoming more stratified (at least in some regions). Temperature directly affects metabolic rates and also indirectly affects organisms due to water column stratification restricting nutrient supplies to the surface (Behrenfeld *et al*., 2006). Warming is also causing pronounced retreat of sea ice in the Arctic Ocean (http://nsidc.org/data/seaice_index/) with severe consequences for the entire polar food web (Smetacek & Nicol, 2005). Finally, oxygen minimum zones at depth seem to be expanding and becoming more intense (Stramma *et al*., 2008), with implications for ocean N, P and Fe cycles and thus the balance of nutrient limitation in the sea (Moore *et al*., 2013). However, recent research indicates that anoxia in the North Pacific can be linked to tropical trade winds and if they become weaker as predicted, the ocean's largest anoxic zone will contract despite a global O_2_ decline (Deutsch *et al*., 2014).

We know that life can adapt to changing environmental conditions by individual organisms migrating or altering their growth strategies (acclimating) and by populations adapting over time through genetic (or epigenetic) evolution. On land, long‐lived plants that account for 50% of global primary production may struggle to evolve as fast as the climate changes. In the ocean, in contrast, where most of the primary producers are either single‐celled microbes or fast‐growing macroalgae, there is considerable potential for them to evolve rapidly to changing environmental conditions (Lohbeck *et al*., 2012). Warming is considered to be a strong selective agent that is likely to drive evolutionary change in most taxa (Thomas *et al*., 2012; Boyd *et al*., 2013). However, our knowledge of how marine microbes may acclimate and evolve in a changing ocean is fundamentally incomplete, and most existing models (e.g. Le Quere *et al*., 2005; Follows *et al*., 2007) fail to consider adaptive responses. There is thus an urgent need to improve our understanding of how marine ecosystems and their constituent organisms respond to environmental change and how these responses in turn feedback to affect the magnitude of environmental change.

**Figure 1:**
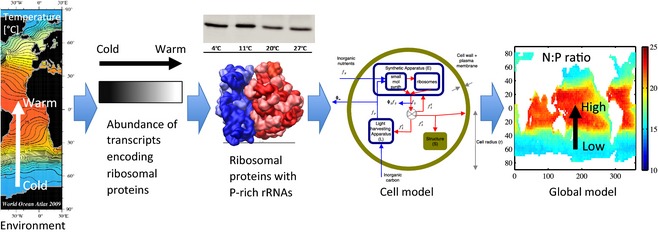
Informing the Earth system science with marine phytoplankton by omics data. Metatranscriptome sequences from natural phytoplankton communities helped to identify physiological traits (cellular concentration of ribosomes and their rRNAs) underpinning adaptation to environmental conditions (temperature). A mechanistic phytoplankton cell model was used to test the significance of the identified physiological trait for cellular stoichiometry. Environmental selection in a trait‐based global marine ecosystem model was then linking emergent growth and cellular allocation strategies to large‐scale patterns in light, nutrients and temperature in the surface marine environment. Global predictions of cellular resource allocation and stoichiometry (N:P ratio) were consistent with patterns in metatranscriptome data (Toseland *et al*., 2013) and latitudinal patterns in the elemental ratios of marine plankton and organic matter (Martiny *et al*., 2013). Three‐dimensional view of ribosome was taken from Wikipedia, showing rRNA in dark blue and dark red. Lighter colours represent ribosomal proteins. Bands above show temperature‐dependent abundance of the eukaryotic ribosomal protein S14, adapted from Toseland *et al*. (2013).

A key connection that needs to be strengthened is between the insights into the marine microbial ecosystem coming from molecular biological omics data (Hood *et al*., 2006) and existing biological, ecological and modelling approaches to studying the impact of environmental change on marine organisms (Fig. 1). Omics studies have revolutionized our understanding about how organisms have evolved and are adapted to environmental conditions of the oceans. Nucleic acids record both how the environment affects organisms and how organisms respond to changing environmental conditions. They thus offer a repository of information that has yet to be fully integrated into current understanding of the structure and functioning of marine ecosystems.

Crossing the scales from omics science to the marine ecosystem response to environmental change requires several intermediate steps (Fig. 1). Here, we argue that the framework of evolutionary ecology – drawing on systems biology, physiological measurements and experimental evolution studies – can help provide that bridge. A central concept is that organisms and their fitness‐determining phenotypic traits have been optimized by natural selection. Thus, if we can capture the key traits of marine microbes, the trade‐offs between them and the environmental selection pressures on them, we can understand the emergence of successful phenotypes. A key opportunity opened up by omics science is to underpin this phenotypic‐level understanding with new knowledge of the underlying genetics and biochemistry. Systems biology can help here by mapping from genes and biochemistry to the costs and benefits of maintaining key components of the cell. Physiology provides empirical measurements to test this understanding of cellular economics, and experimental evolution studies provide information on the possibilities for, and constraints on, adaptation.

Here, we address how integrating omics approaches and evolutionary ecology into our models of the marine ecosystem could lead to a step change in our understanding of how environmental change impacts marine organisms and the challenges this raises. We focus on marine microbes as the application of omics sciences in the marine system is at the forefront for this group of organisms. Furthermore, marine microbes such as phytoplankton and heterotrophic bacteria have a significant impact on marine food webs and biogeochemical cycling, which is why comprehensive data sets are now available from omics to modelling. Thus, marine microbes provide an ideal test case for developing new integrative ecosystem approaches that address some of the most significant challenges human race and societies have ever faced.

The rest of the article is structured as follows. First, we consider how omics approaches have revealed new insights into the ocean's secrets by identifying the outcome of millions of years of evolutionary adaptation of microbes. Second, we discuss how changes in the surface oceans challenge the evolutionary adaptation of marine microbes. Third, we consider how we can advance our understanding of climate‐driven changes in the oceans through using new models that integrate knowledge from omics approaches with fundamental concepts of evolutionary ecology.

## Omics approaches: revealing the ocean's secrets

### Drivers of microbial biogeography

Omics approaches have enabled us to add an organism‐centric view to the Earth system science. In the past, geochemists often identified biogeochemical processes before biologists identified the responsible organisms. Now omics is identifying a plethora of organisms that may be responsible for a wide range of energetically feasible biogeochemical processes. Marine microbes were among the first targets for genome‐enabled science (Dufresne *et al*., 2003; Rocap *et al*., 2003; Derelle *et al*., 2006). Studies on ribosomal genes such as 16S or protein‐coding marker genes provided the first step towards a taxonomic census of marine microbes in their environments (Schmidt *et al*., 1991). Remarkable diversity has been observed for most of the different marine microbial groups. As natural historians have mapped the distribution of animals and plants on land, marine microbial scientists are now able to map marine microbes on a global scale for insights into their biogeography (Follows *et al*., 2007), which is the first step to understanding how the environment shapes microbial diversity in the oceans. Several biogeographical studies based on metagenome sequences revealed that many heterotrophic and autotrophic marine microbes show divergence into phylotypes specifically adapted to either different oceanographic provinces or lifestyles (e.g. Dinsdale *et al*., 2008). Those phylotypes are referred to as ecotypes because they are adapted to specific environmental conditions representing traits that reflect most successful adaptations to a given environment.

Interestingly, the most fundamental and significant driver of global microbial diversity in the surface oceans seems to be temperature (e.g. Raes *et al*., 2011; Thomas *et al*., 2012; Toseland *et al*., 2013). Marine bacterial diversity peaks globally at high latitudes in winter (Ladau *et al*., 2013). This pattern strongly contrasts with tropical, seasonally consistent diversity peaks observed for most marine and terrestrial macro‐organisms (Hillebrand, 2004). There is also evidence that human impact significantly increases bacterial diversity in surface oceans as global hot spots include coastal waters and the Arctic Ocean, both of which are significantly impacted by river run‐off and human activity (Ladau *et al*., 2013). Besides temperature, another strong predictor of bacterial richness is day length (Gilbert *et al*., 2012), which explains the seasonality of diversity in temperate oceans. Nutrients seem to play a smaller role in determining global diversity patterns of marine microbes, but among the nutrients, phosphate has the strongest predictive power of heterotrophic bacterial diversity (Ladau *et al*., 2013). Biogeographical controls on marine nitrogen fixers seem to be controlled by low fixed nitrogen and sufficient iron and phosphate (Monteiro *et al*., 2011). Higher temperature requirements, quite often used to explain their biogeography, seem to be adaptations to these particular environments and, therefore, not primarily controlling their distribution (Monteiro *et al*., 2011).

Another strong predictor of bacterial diversity in the ocean is water depth, which is unsurprising as there are strong vertical gradients in (i) light quantity and quality, (ii) temperature, (iii) pressure and (iv) general environmental variability. Several studies give evidence of depth‐specific microbial communities with strong separations between the photic and aphotic zones (e.g. Ghiglione *et al*., 2012). Even at the poles where the oceans are subjected to strong vertical mixing and upwelling, there was a difference in the estimated diversity between surface and deep microbial communities (Ghiglione *et al*., 2012). A higher degree of diversity was observed in the deep ocean compared to the surface ocean based on the V6 region of the SSU rRNA gene (Ghiglione *et al*., 2012). However, by taking into account the geographical differences in bacterial communities of the surface oceans across latitudes, it seems that surface communities differ more than deep communities. Environmental drivers in the deep ocean may be weaker compared to surface waters because several factors including temperature are more uniform in the deep ocean. There is also evidence that deep communities are more connected through oceanic circulation, which might explain why these communities differ less between the Arctic and Southern oceans. Both oceans are connected by deep bottom currents that transport microbes across the equator. In contrast, the majority (85%) of polar surface microbial communities appeared to have pole‐specific distributions, suggesting incomplete dispersal due to geographical isolation (Ghiglione *et al*., 2012).

All of the phylogenetic assessments of microbial diversity in the oceans so far have revealed evidence for biogeographically defined communities. It seems that these communities evolved according to distinct environmental conditions. Positive selection seems to be the underlying driver of ecological niche differentiation, highlighting the existence of genomic traits characteristic of different phylotypes thriving in specific marine biomes.

### Environmental adaptation of microbial metabolism

Adaptive radiation of marine microbes in different marine biomes is underpinned by metabolism reflecting traits characteristic for these biomes (Dinsdale *et al*., 2008). Comparative metagenomics of microbial communities from different marine biomes has revealed a first glimpse into how the environment shapes metabolism of these microbes and, therefore, their evolution and radiation. Interestingly, it seems that the biogeographical patterns of species diversity are mirrored in metabolic differences reflecting molecular functional traits such as nutrient requirements in relation to the number and diversity of nutrient transporters (Patel *et al*., 2010). Thus, environmental parameters used as predictors of species diversity also serve as predictors of molecular trait diversity. Orthologous groups (OGs) of genes have been used as a measure of molecular functional richness in a metagenome of a microbial community. The number of OGs in relation to the evenness of the functional distribution reflects the diversity of the metabolic potential of a community similar to rRNAs reflecting the taxonomic diversity. A detailed analysis of OGs from the GOS data set revealed that most of the metabolic diversity in microbial communities from the surface ocean can be explained by temperature and light, very similar to the species diversity based on rRNA genes. Furthermore, a significant negative correlation between the functional diversity and primary productivity was observed for functional richness. This observation supports global taxonomic data that showed highest bacterial diversity in winter in temperate oceans when primary productivity is at its annual minimum.

A study focussed on membrane proteins in the GOS data set revealed how closely the environment impacts the abundance of functional protein groups and thus selects traits. Membrane proteins play a fundamental role in sensing and interacting with the environment but also in terms of energetics as photosynthesis and respiration are membrane‐bound processes. Thus, membrane proteins are ideal to test how the environment shapes microbial metabolism and adaptation. Patel *et al*. (2010) developed an environmental features network to quantify correlations between protein families and covarying environmental features. They showed that specific protein families are enriched under specific environmental conditions. For instance, the affinity of phosphate transporters from the GOS data set was related to the concentration of phosphate in the environment, whereas the occurrence of iron transporters was connected to the amount of shipping, pollution and iron‐containing dust deposited in the surface ocean (Patel *et al*., 2010). Thus, those proteins have the potential to be used as *in situ* biomarkers and, therefore, as tools to assess how environmental change impacts microbial communities in the ocean. However, more important than the presence of genes in a given microbial community is their activity measured either by transcript or protein abundance (e.g. Toseland *et al*., 2013; Saito *et al*., 2014; Alexander *et al*., 2015). Several studies have already shown potential for detecting *in situ* biomarkers specifically for nutrient stress by measurements of upregulated transcripts or proteins associated with nutrient stress (e.g. Lindell & Post, 2001; Webb *et al*., 2001; Marchetti *et al*., 2012; Chappell *et al*., 2015). Furthermore, a recent study by Saito *et al*. (2014) revealed how quantitative mass spectrometry‐based protein biomarker measurements can be used to characterize nutrient limitation patterns for multiple nutrients on the abundant cyanobacterium *Prochlorococcus*. Targeting several different biomarkers for nutrient stress (e.g. IdiA for iron stress, P‐II for nitrogen stress) simultaneously across a meridional transect in the central Pacific Ocean revealed widespread and overlapping biogeochemical regions of nutritional stress for nitrogen and phosphorous in the North Pacific Subtropical Gyre and iron in the equatorial Pacific. Furthermore, quantitative protein analysis demonstrated simultaneous stress for these nutrients at biome interfaces, which had not been observed before with other methods. These data are promising and contribute to translational applications from cell biology to be applied to assessing the state of marine microbial communities under global change. Similar translational approaches are currently being applied to conservation efforts for coral reefs (Traylor‐Knowles & Palumbi, 2014). However, a major weakness seems to apply to both fields: we still lack understanding of some of the basic mechanisms underpinning the regulation of biomarkers. As long as biomarkers have only been developed for specific species (e.g. cyanobacteria, diatoms and corals) without fully understanding their mechanisms or genotype or acclimatization ability, their use is limited as a large amount of variation will be left unexplained (e.g. Traylor‐Knowles & Palumbi, 2014). To tackle this issue, we need to determine the mechanisms by which the biomarker is being activated and controlled by applying techniques from cell biology to ecological key taxa underpinning biogeochemical processes.

Those mechanistic insights together with physiological data will give clearer evidence for environmental adaptation of microbial metabolism and lay the foundation for trait‐based modelling.

Microbial community metabolism also differs with seawater depth as shown by a metagenomics study at the Hawaiian Ocean Time‐series (HOT) station (Delong *et al*., 2006). OG analysis identified metabolic differences between photic and aphotic communities reflecting differences in microbial diversity. Most of the sequences from the photic zone were involved in photosynthesis, iron transport, efflux pumps and membrane proteins, whereas transposases, pilus synthesis proteins, protein export, polysaccharide and antibiotic synthesis were mostly enriched in deeper waters. The sequences from deep‐water communities give some evidence for a surface‐attached lifestyle that may be related to life in aggregates of organic matter.

Taken together, taxonomic and functional metagenomics, meta‐transcriptomics and quantitative meta‐proteomics studies of marine microbial communities have revealed that environmental conditions determine taxonomic and functional diversity in the same direction across different biomes from poles to the tropics, between different nutrient regimes and vertically from the surface to the deep ocean. The consequence is selection of those traits that are most successful under given environmental conditions. These conditions although vary temporally and spatially leading to the evolution of different phylotypes (ecotypes) selected under specific environmental conditions. However, undeniably, there are still major gaps and limitations in our understanding of how microbes are adapted to their natural environment, which might limit our ability to construct metabolic networks underpinning trait‐based modelling. We will begin to close these gaps if we couple knowledge from cell biology with integrated outputs from various omics approaches mentioned above. To the best of our knowledge, there are no studies so far on marine microbes and their communities that extend all the way from genomics to physiology in a single coherent study. But only with these integrated approaches will we be able to understand the evolution and regulation of functional traits and therefore improve current biogeochemical models.

### The genetic basis of adaptation and metabolism

Marine microbes are prone to fast evolution as they usually have large census population sizes, so that mutations on which natural selection can act arise often. In populations that are far from their optimum in the fitness landscape, there is a higher proportion of advantageous mutations that confer increases in fitness in larger as compared to smaller populations (Luo *et al*., 2014). Advantageous mutations become fixed faster and spread more quickly through a larger population (Lanfear *et al*., 2014). However, the substitution rate depends on both the rate at which new advantageous mutations occur in a population and the time that each mutation spreads to fixation. Nevertheless, it seems adaptive evolution under changing environmental conditions, where locally adapted populations may experience drops in fitness, will be faster in larger populations if the mutation rate is high. Under varying dynamic environmental conditions with large spatial and temporal variability, genotype sorting within diverse species is likely if there is variation in fitness within that species (Lohbeck *et al*., 2012; Schaum *et al*., 2013). There is evidence of intraspecies variation in fitness, where different populations of the same species alternate in abundance in time (e.g. seasonal succession) or space (e.g. latitude) depending on their fitness peak in relation to environmental conditions.

This is exemplified by ecotypes of the cyanobacterial genus *Prochlorococcus* (Johnson *et al*., 2006; Kashtan *et al*., 2014). Here, the same intraspecies variation that allows the maintenance of diversity in fluctuating environments could be used as fuel for directional selection if the nature of environmental variation changes. *Prochlorococcus* can be divided in several different ecotypes with distinct seasonalities, depth distributions and geographical locations. For instance, there are high‐light‐adapted and low‐light‐adapted ecotypes, and ecotypes that prefer warmer water and those that occur in colder waters at higher latitudes (Johnson *et al*., 2006). They can be identified both by their rRNA genes and differences in their gene composition. Also for *Prochlorococcus*, temperature and light seem to be the most important environmental variables shaping their diversity (Johnson *et al*., 2006). About 26% of variability in the total *Prochlorococcus* population studied in the Atlantic Ocean could be explained by temperature (Johnson *et al*., 2006). One of the two high‐light‐adapted strains (eMED4) was more abundant at higher latitudes (30–50°) because it could grow between 10 and 15°C, whereas eMIT9312 was more abundant at low latitudes because it stopped growing at around 15°C. A whole‐genome comparative analysis between these ecotypes revealed the existence of strain‐specific differences in five major genomic islands (GI), which had been acquired via horizontal gene transfer from other bacteria, archaea and/or phages (Coleman *et al*., 2006). The five islands in eMED4 and eMIT9312 were located at the same position in both genomes and therefore were considered hot spots of recombination. Some of them showed signs of remodelling by the presence of repeats, and up to 80% of the genes in these islands were most similar to genes of non‐cyanobacterial organisms, indicating horizontal gene transfer. However, how the genes in these islands are involved in adaptation to different environmental conditions and thus niche separation remains enigmatic. Nevertheless, there is clear evidence from many more cyanobacterial genome sequences that GIs enable local niche adaptation and are therefore crucial to understand global biogeography in cyanobacteria. Thus, these GIs confer new characteristics to the organisms allowing them to jump from peak to peak within the fitness landscapes, a characteristic that is similar to other microbes from very dynamic environments (e.g. gut microbiota) (Ley *et al*., 2006; Juhas *et al*., 2009).

How this adaptation potential is realized in eukaryotic marine microbes is still very elusive. However, the first genomes from eukaryotic phytoplankton have revealed, similarly to their prokaryotic counter parts, that the environment significantly impacts genome architecture and gene composition. For instance, horizontal gene transfer in prasinophytes and diatoms is thought to have facilitated species divergence (Derelle *et al*., 2006; Bowler *et al*., 2008). While the prasinophyte green alga *Ostreococcus* has acquired two complete chromosomes via horizontal gene transfer, HGT in diatoms so far seems to be restricted to single genes from bacteria, archaea or fungi. These alien genes, both of bacterial origin, facilitate niche separation in diatoms as shown for rhodopsins (Marchetti *et al*., 2012) and antifreeze proteins (Raymond & Morgan‐Kiss, 2013) in diatoms. The function of the alien chromosomes in *Ostreococcus* remains more enigmatic as most of their genes have unknown function, and no functional characterization through reverse genetics approaches has been published so far (Derelle *et al*., 2006).

Species‐specific transcriptomics, proteomics and metabolomics studies with marine microbes provided first insights into the significance of single genes or gene clusters for acclimation and adaptation of marine microbes to environmental conditions (e.g. Mock *et al*., 2008; Allen *et al*., 2008; Wecker *et al*., 2009; Zinser *et al*., 2009, Ashworth *et al*., 2013, McKew *et al*., 2013). Furthermore, those studies laid the foundation for biomarker discoveries to study natural communities. Some of the earliest targets for those studies were marine heterotrophic bacteria, cyanobacteria and diatoms. Most of these omics studies revealed that the species tested were highly responsive to changes in their environmental conditions (e.g. Allen *et al*., 2008; Mock *et al*., 2008; Wecker *et al*., 2009; McKew *et al*., 2013). Furthermore, metabolic pathways responsible for acclimation to environmental conditions could be identified even for species in natural communities revealing how metabolism can differ between species from the same environment (Alexander *et al*., 2015). These studies are invaluable for subsequent physiological and biochemical measurements underpinning trait‐based modelling. However, as far as we know, none of the published studies with marine microbes has applied omics approaches yet to measure the physiological response on evolutionary time scales (≥200 generations), which is a major gap for identifying those genes, promoters and transcripts that are under positive selection and, therefore, responsible for coping with changing environmental conditions. Furthermore, the role of epigenetics for acclimation and adaptation of marine microbes still is very enigmatic as only very few studies have addressed the role of epigenetics (Veluchamy *et al*., 2013). However, as epigenetic changes might significantly contribute to the plasticity of the phenotype (Schlichting & Wund, 2014), epigenetics might hold great promise for understanding the dynamics of physiological responses.

Thus, a key question that remains to be answered is how fast and how dynamically marine microbes can evolve under changing environmental conditions. Here, a recent study on single‐cell genomics with wild *Prochlorococcus* populations is leading the way in answering that question (Kashtan *et al*., 2014). A cell‐by‐cell comparison between co‐occurring populations of *Prochlorococcus* revealed that these communities were composed of hundreds of subpopulations with distinct ‘genomic backbones’, each backbone consisting of different sets of core alleles linked to a small set of flexible genes typically in form of cassettes within genomic islands. The genetic variation between backbone subpopulations of *Prochlorococcus* is explained by the population structure as the fixation index FST (a measure of population differentiation due to genetic structure) is between 0.8 and 1.0, indicating almost complete subpopulation separation. The different backbone subpopulations seem to have different niches (which are separated in time) as the relative abundances of subpopulations changed according to environmental conditions (autumn, winter and spring). Due to the enormous population size of these backbone subpopulations (>10^13^ cells), it is likely that they have evolved by selection. Moreover, the backbone subpopulations maintained their genomic composition between seasons, which supports the Baas‐Becking hypothesis that ‘everything is everywhere but the environment selects’ (Baas‐Becking, 1934). Rarefraction analysis of these backbone subpopulations that coincided well with ITS ribotypes revealed at least hundreds of subpopulations co‐occurring at the same time and location but with differential abundance according to environmental conditions (seasonality) (Kashtan *et al*., 2014). They were estimated to have diverged at least a few million years ago, suggesting ancient niche partitioning. The extant populations though seem to reflect a stable and balanced collective of ecotypes that may refine their gene repertoire only slightly due to the lack of strong selection pressure.

Based on the population study with *Prochlorococcus* (Kashtan *et al*., 2014) together with the evidence for horizontal gene transfer described by Coleman *et al*. (2006), it appears that diversity is initially generated when there is strong selection pressure due to rapid and significant environmental change that might drive the exchange of genetic material via HGT, which seems to lead to large fitness gains over a short period of time similar to other dynamic environments where the same strategy has been established (gut microbiota) (Ley *et al*., 2006). However, once stable niche partitioning has taken place, subpopulations are maintained by environmental fluctuations, and within‐subpopulation evolution is constrained to mutations and natural selection, which refines the fit of subpopulations to their niche.

## Adaptation to environmental change

The response of marine microbes and microbial communities to environmental change depends on both the magnitude and the time scale of the change. On very short time scales, cellular physiology can respond rapidly to changes in resource availability (e.g. light and nutrients) or physical/chemical stressors (e.g. low or high temperature, ocean acidification, UV radiation). On slightly longer time scales of hours to days, cells and cell populations can acclimate by changing their phenotypes through synthesis and degradation of macromolecules. On longer time scales of days to months, microbial communities can be remodelled as dominance patterns within the community change or species are introduced to or lost from the local environment. On longer time scales, populations may evolve through natural selection.

### Adaptation at the cellular level

On the level of a microbial cell, ocean acidification is considered to impact the pH homoeostasis (Taylor *et al*., 2012) and therefore impact many enzymatically regulated physiological processes such as nutrient uptake, osmoregulation, photosynthesis and calcification (Bach *et al*., 2012). pH may operate directly or indirectly via changes in the inorganic carbonate system by changing the concentrations of carbon dioxide, bicarbonate or carbonate ions and the saturation states of aragonite and calcite in seawater (Plummer & Busenberg, 1982).

Warming is considered to impact the overall temperature‐dependent metabolism (Arrhenius equation). Enzyme kinetics are strongly dependent on temperature (Q_10_ = 2–3), and therefore, many reactions involved in resource allocation (e.g. nutrient uptake, peptide elongation, fatty acid synthesis, and TCA cycle) are affected by changing temperatures (Raven & Geider, 1988). In contrast, the Q_10_ for light absorption by chlorophyll = 1.0 (Raven & Geider, 1988), so in the absence of acclimation, changing temperatures can lead to a decoupling of the potentials for ATP/NADPH production and carbon fixation in autotrophs, such as phytoplankton. Thus, temperature has a significant impact on the energetics of individual cells. Many cellular signalling and regulatory pathways are also affected, both directly by temperature and in response to metabolic changes. For example, the imbalance between energy supply by temperature‐independent light absorption and energy consumption by temperature‐dependent enzymatic reactions is sensed in the chloroplast by modulation of the redox state of the photosynthetic apparatus. This redox information is conveyed to the nucleus affecting gene expression and leading to remodelling of the photosynthetic apparatus to re‐establish an energy balance (Hüner *et al*., 2012). Molecular studies offer the opportunity to understand the mechanism of these intracellular changes, which constrain plasticity. Temperature responses remain to be investigated at the level of molecular networks, largely because the candidate cellular pathways are still being elucidated, including photoreceptors (Coesel *et al*., 2009; Huysman *et al*., 2013), the circadian clock (Corellou *et al*., 2009; O'Neill *et al*., 2011), cell cycle regulators (Moulager *et al*., 2007), protein kinases (Hindle *et al*., 2014) and starch metabolism (Ral *et al*., 2004; Sorokina *et al*., 2011). However, molecular responses to ambient temperature are a topic of current research in systems biology, including in higher plants (Franklin *et al*., 2014), where light response pathways closely interact with ambient temperature signalling (Gould *et al*., 2013). This work builds on research in *E. coli*, yeast and fruit flies (Bochdanovits & De Jong, 2003; Bennett & Lenski, 2007; Piotrowski *et al*., 2012). All this work showed that temperature has a significant impact on the organization of genomes and that resistance to heat has a genetic origin.

### Adaptation at the level of populations

How current climate change impacts the evolution of microbial communities remains to be seen, but time will tell as human race has already begun a selection experiment on a global scale. Marine microbes may already be well prepared to respond appropriately with genes or GIs that will be exchanged again in a period of rapid change. Alternatively, the current genetic variation may not be sufficient to allow marine microbes to cope with climate change. Thus, current environmental change might push marine microbes out of their environmental envelope of the past several million years, in which case the rise of new beneficial mutations may be used instead of or in addition to sorting existing variation, although the relative importance of genotype sorting and selection on *de novo* variation in microbial populations has yet to be established. Temperature appears to have significantly shaped the current large‐scale microbial diversity in the oceans (Johnson *et al*., 2006; Raes *et al*., 2011; Thomas *et al*., 2012; Toseland *et al*., 2013), and it is temperature change that is one of the major consequences of the anthropogenically induced climate change.

Phenotypic plasticity (the ability of a single genotype to produce multiple phenotypes), phenotypic diversity (the number of phenotypes with different fitness present in a population) and the population size determine how a population is able to respond to environmental change in the short term by genotype sorting without contributions from *de novo* mutation (Via & Lande, 1985). Over the longer term, mutational supply (mutation rate x effective population size) is also important in generating novel heritable variation in fitness on which natural selection can act. Different species of marine microbes differ in their degree of phenotypic plasticity (Schaum *et al*., 2013), and taxa are thought to differ in the amount of genetic and phenotypic diversity present, although little empirical data on which to base comparisons exist. The molecular and modelling tools are now established to understand such natural variation at the mechanistic level, at least for some molecular systems (Monnier *et al*., 2010; Troein *et al*., 2011). It is unknown how marine microbes differ in their ability to respond evolutionarily to environmental change such as ocean acidification and warming, because this requires taking into account *de novo* mutation as well as evolutionary constraints – both of which need to be investigated empirically (for a recent review, see Collins *et al*., 2013).

### Adaptation at the community level through range shifts

Recent studies reveal that warming of the surface ocean is responsible for a significant poleward range shift in dispersal of marine pelagic organisms including plankton (Poloczanaska *et al*., 2013). Indeed some phytoplankton and zooplankton species show the highest velocity in range shift dispersals (>400 km/decade) (Poloczanaska *et al*., 2013). Those species probably remain in the same thermal niche while they are shifting polewards because the niche shifts due to global warming. However, poleward‐shifting thermal niches are not identical replicates of their geographical origin at lower latitudes as warming affects mixing and thus nutrient availability. Furthermore, poleward‐shifting marine organisms experience changing seasonality of solar irradiance (day length and irradiance) depending on the latitude. Thus, for successful range shift dispersals of populations, species with wide tolerance ranges will most likely be at an advantage. Wide tolerance ranges in general are underpinned by adaptive plasticity that is favoured by strongly fluctuating environments such as temperate ecosystems (Davis & Shaw, 2001). In contrast, those species that have a very limited range of dispersal due to specific adaptiveness are most sensitive to climate change. Polar and tropical ecosystems harbour many different communities of organisms that are adapted to a relatively narrow temperature range and therefore have limited dispersal (Boyd *et al*., 2013). If temperature deviates even slightly from the annual average temperature, it will affect the diversity and productivity of these communities (Hof *et al*., 2012; Poloczanaska *et al*., 2013).

Significant poleward range shifts in dispersal of marine plankton and increasing extinction rates for those organisms with a narrow range of adaptation such as polar and tropical species show that global warming impacts the largest ecosystem on the Earth (Davis & Shaw, 2001). Without knowing the evolutionary potential of key players in marine ecosystems, we cannot reliably predict future responses to global warming especially for those organisms with a low tolerance ranges and thus limited adaptive capacity.

### Adaptation at the community level through selection

Most species studied have an optimum temperature and pH for growth and reproduction and show evidence of decreased fitness when grown under nonoptimal conditions. Furthermore, comparative studies often show that populations are adapted to their temperature regimes (Boyd *et al*., 2013), although evidence is equivocal for adaptation to local CO_2_ (Langer *et al*., 2009; Lohbeck *et al*., 2012; Hutchins *et al*., 2013). The paucity of data on specific carbonate system regimes might be one reason why there is insufficient evidence for phytoplankton to be selected by past environmental conditions to occupy different CO_2_ niches. A lack of fit between extant populations and the new environment could be restored by directional selection that increases the prevalence of genotypes with adaptive traits that are better suited to the new conditions.

Selection in new environments favours genotypes that are better suited to the new external conditions (i.e. can survive and reproduce better). Fitter organisms may be present at low abundance within endogenous populations and communities, or they may invade to displace less fit residents under the new conditions. Large populations made up of individuals who reproduce quickly (e.g. bacteria and phytoplankton) can often adapt rapidly to new conditions because they have a higher supply of beneficial existing variants and high standing variation, which natural selection can act on (Elena & Lenski, 2003). They can often also respond through phenotypic plasticity to changing environments, which further increases their chances of evolving (Draghi & Whitlock, 2012). That said, physical (e.g. dissolution rates of calcite), genetic (e.g. pleiotropy) and historical constraints may limit the evolutionary potential of taxa even in the face of high mutational supplies.

### Evolution of ecosystem function

We know that ocean acidification and global warming significantly affect the diversity of communities with propagating effects for food webs and biogeochemical cycles and that changes in community composition are the consequence of a lack of fit between endogenous populations and the new environmental conditions (Davis & Shaw, 2001). A key to linking changes in ecosystem function to evolution of key organisms in the face of global change is to understand which traits evolve in those organisms and how these traits affect individual fitness under relevant environmental conditions. Understanding the differences in how key taxa evolve in response to ocean acidification and associated warming will substantially improve predictions of how marine ecosystems and ecosystem services are likely to change in response to global environmental change.

## From microbes to ecosystem‐level properties

Understanding the ecological basis for the observed ecosystem‐level properties requires comparison of theoretical models for organism distribution and function (either based on traits and niche modelling, or fully mechanistic ‘dynamical system’ models) with observations. Understanding of organism properties has been derived from laboratory measurements of ecophysiology, usually focussing on limiting nutrients and light as controls on phytoplankton growth rates (Boyd *et al*., 2010), supplemented by available *in situ* measurements including nutrient addition experiments (Moore *et al*., 2013) and photophysiology. Empirical understanding of ecosystem structure and function has been based on correlative studies between (i) ocean measurements of environmental parameters including temperature, nutrient levels and light (World Ocean Atlas); (ii) *in situ* ‘inventory’ measurements, both bulk measurements of plankton biomass, C/N/P *in situ* and remote sensing of chlorophyll and pigments (Buitenhuis *et al*., 2013), and taxonomic classification; (iii) *in situ* ‘rate’ or ‘flux’ measurements including growth rates and primary production (Juranek & Quay, 2013; Laws, 2013), predation rates (Buitenhuis *et al*., 2006, 2010) and export production via sediment traps.

Theoretical models have employed three main approaches. Approaches based on traits and niche modelling provide a framework to link to ecological theory (Litchman & Klausmeier, 2008) and are able to interpret bottom‐up controls (light, nutrients, temperature) on phytoplankton biogeography (Litchman & Klausmeier, 2008; Barton *et al*., 2013). Approaches based on general circulation model (GCM) representations of the ocean environment and biogeochemical cycles via parameterizations of ecosystem function primary production, nutrient recycling and export) as a function of environmental parameters are able to capture major features of nutrient distribution and nutrient and carbon fluxes (Ridgwell *et al*., 2007). Mechanistic ecosystem models (recently reviewed by Follows & Dutkiewicz (2011)) incorporate aspects of both these approaches and can account both for (i) bottom‐up controls of phytoplankton community composition and structure as a consequence of environmental selection of the fittest taxa, and (ii) the influences of biotic interactions and ecosystem feedbacks to biogeochemistry. These have typically employed ‘black‐box’ representations of organism ecophysiology based on parameterization of a small number of traits for nutrient acquisition, light acquisition, temperature‐dependent growth rate and grazing interactions – an approach pioneered by Riley (1946). More recent approaches dramatically improve representation of the global environment via GCMs and have started to address biological diversity either explicitly as traits, or via representation of plankton functional types (PFTs) (Le Quere *et al*., 2005). Heterotrophic recycling, however, has still usually been parameterized, via rates of remineralization of particulate and dissolved organic pools.

All these approaches agree on major features and controls on present biogeography: top‐down controls are of major importance, with the global mean ratio of export to primary production ~ one‐third. Permanently stratified low latitudes favour small phytoplankton, strategies based on resource competition, and show highly efficient nutrient recycling via the microbial loop. Seasonally stratified high latitude favours larger phytoplankton with relatively high export production.

The overall bottom‐up controls on phytoplankton at low latitudes are reasonably well represented and understood in terms of (nutrient) resource competition theory and light availability (Follows *et al*., 2007). However, detailed understanding of vertical structure in permanently stratified regions requires detailed consideration of additional traits for irradiance spectra and hence pigment‐dependent light harvesting strategies and photoprotection (Hickman *et al*., 2010). Mixotrophy is also potentially important (Hartmann *et al*., 2012).

High latitudes and bloom‐forming taxa (diatoms, coccolithophores) are not well represented in current models (Vogt *et al*., 2013) suggesting complex trait interactions (Hashioka *et al*., 2013), or maybe missing key traits, for example the importance of fluctuating light environments (Talmy *et al*., 2013), iron–light tradeoffs (Behrenfeld & Milligan, 2013), resting/survival strategies or armour/defence strategies (Behrenfeld & Boss, 2014). In particular, the discrepancy between the high (~40%) diatom contribution to global export production inferred from biogeochemistry (via silica fluxes) and the small apparent areas of diatom dominance in satellite observation, combined with limited success in model‐based prediction of diatom distribution, suggests that a more detailed understanding of ecosystem structure is required (Vogt *et al*., 2013).

Given that marine biogeographical ‘provinces’ can be identified based on combinations of environmental conditions, the response to a changing environment could be viewed as a spatially shifting biogeography and/or as changes within biogeographical provinces. However, the response of the very different systems in oligotrophic low latitudes and in nutrient‐rich high latitudes could be quite different under the same predicted environmental change (Doney, 2006).

Traits and niche modelling provides a framework to understand the ecological response to environmental change, which they represent via species sorting (Litchman *et al*., 2012; Thomas *et al*., 2012; Edwards *et al*., 2013). Mechanistic models support the view that the largest effects of environmental change may be on community composition (Dutkiewicz *et al*., 2013).

Mechanistic models can additionally include feedbacks to biogeochemical cycles. These models demonstrate that export production is robustly linked to nutrient supply to the well‐mixed surface layer – which is expected to decrease overall in a warming and stratifying ocean. However, there are potentially three compensating effects on global primary productivity – an overall reduction in primary productivity due to reduction in nutrient supply, versus an increase in growth rate due to temperature (Taucher & Oschlies, 2011), versus a CO_2_ fertilization of growth that might help to offset lower primary production under more stratified conditions in a warm ocean (Oschlies, 2009). There is some agreement between models on general patterns with predicted reductions in primary production in the stratified subtropics, but increases in the Southern Ocean as light and temperature limitation is alleviated (Marinov *et al*., 2010). CMIP5 model responses agree on an overall forecast decrease in primary productivity but are quite disparate regarding its magnitude (Bopp *et al*., 2013).

Environmental variability provides a potential testing ground of such predictions via ‘natural experiments’ due to interannual or decadal scale variability. Variability at low latitudes is dominated by ENSO and strongly perturbs primary productivity (Behrenfeld *et al*., 2006) and also affects community composition (Masotti *et al*., 2011). Variability at high latitudes is dominated by the annular modes (SAM, NAO) where changes in wind‐driven mixing drive changes in diatom abundance (Alvain *et al*., 2013).

However, all of these approaches are grounded in empirical descriptions of organism traits and, hence, fundamentally limited in predictive power by the accuracy of process‐based understanding. None of them can include the adaptive response to novel environments (other than via further parameterization of direct measurements), nor can they be related to omics data sets. Thus, we currently lack confidence in using current models and modelling frameworks to make projections of future ecosystem structure and function under new environmental conditions that will lie outside the historical envelope.

## Making predictions: bringing subcellular processes to the global scale

**Figure 2:**
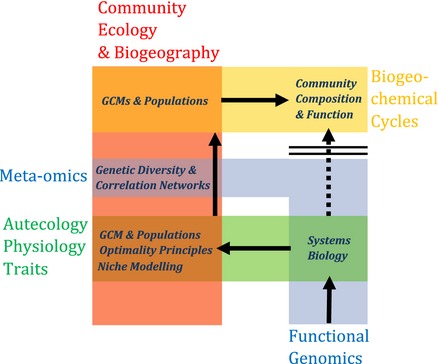
Bridging the gap: a model‐centred approach to integrating omics approaches with marine microbial ecology. Omics approaches (blue bars) provide new insights both at the level of population and community structure (red bar), and into physiology at organism level (green bar) and below. Quantitatively understanding ecosystem structure, function and response to environmental change requires both integration of omics approaches with other methods and a hierarchical forward (or ‘bottom‐up’) modelling approach (blue arrows). This first links omics to physiology via a combination of gene‐scale models (metabolic networks, transcriptional regulation) and whole‐cell models that represent transport processes, storage pools and energetics. It then represents selection in a model environment to predict community composition and function from organism traits. Evaluation against the combination of omics and other data sets (including satellite colour, *in situ* nutrient and rate measurements) then indicates missing processes. Including a model representation of genetic constraints on adaptation (microevolution) derived from laboratory experimental evolution studies and observed genetic diversity and structure then enables a predictive model for response to environmental change.

Given our overview of existing understanding, several key challenges emerge for any modelling approach that hopes to integrate the rapidly developing perspective on marine biology coming from omics research, with physiological and ecological understanding. In particular, four key elements that need to be captured in a ‘next generation’ model are as follows: (i) representation of an omics level view of genes, transcripts and proteins; (ii) representation of the phenomenal biological diversity in the ocean; (iii) representation of acclimation and adaptation to multiple drivers; and (iv) representation of evolutionary constraints (Fig. 2).

Here, we argue that a key gateway to progress will be to change the level of representation in models from organisms (as black boxes) to macromolecular components. This needs to be accompanied by a representation of adaptive responses at the phenotype level as fitness maximization. Furthermore, evolutionary constraints on adaptation need to be captured through empirical parameterization.

The formulation of such a model can then play a key role in enabling interdisciplinary collaboration. This includes (re)integrating physiology and omics in laboratory studies, via systems biology, but with an emphasis on understanding adaptive value as an emergent property of the detailed mechanisms.

### Integrating physiology and omics to provide a trait‐based representation of diversity

A promising recent approach to representing microbial diversity (e.g. Bruggeman & Kooijman, 2007) is based on a generic coarse‐grained physiological model, with traits for organism design (e.g. size) and allocation strategies among a relatively small number of macromolecular components (e.g. biosynthesis machinery, photosynthesis machinery and structural components). The model includes ecophysiological constraints [e.g. diffusive nutrient uptake, (Button, 1998)] and costs, for example both resource allocation to macromolecular components and running costs, including nutrient, energy and reductant budgets (Shuter, 1979; Raven, 1984; Vallino *et al*., 1996). The benefits are then derived as the response to the model environment, represented by a marine general circulation model of the abiotic environment (light, nutrients, temperature) and biotic interactions (e.g. predation, competition, mutualism, commensalism and parasitism).

A problem faced when implementing these models is having an objective way to assign costs and benefits. To date, most such models have used a black‐box approach. Genomics allows these boxes to be defined objectively in terms of a system of metabolic networks and their various regulators. Transcriptomic, metabolomic and proteomic data are used to ascertain how these networks are coupled together and how they are change in response to availability of resources and/or environmental stress. Moreover, systems biology approaches such as flux balance analysis (Steuer *et al*., 2012) provide robust, objective approaches for calculating the capital and running costs (e.g. the materials and energy required to synthesize biomass from inorganic nutrients) of constructing enzymes, pigments and other components. The benefits of changing resource allocations to different metabolic pathways can be quantified by measuring the amounts and catalytic capacity of these components. For example, proteomics can be used to assess changes in the abundances of different metabolic pathways within a species in response to growth under different environmental conditions (Le Bihan *et al*., 2011; McKew *et al*., 2013).

The availability of sequenced genomes of bacteria, cyanobacteria and microalgae has allowed genome‐scale metabolic models to be developed (Kim *et al*., 2012). These genome sequences map the possible reactions that link resources acquired from the environment to the potential for synthesis of macromolecules. When coupled with information on the biochemical composition of biomass and growth rate, the steady‐state fluxes through the reactions that make up the metabolic network can be calculated (Steuer *et al*., 2012). The output is often represented as a flux map. Flux balance analysis (FBA) is a systems biology approach that is of particular relevance to understanding the energetic and capital costs of microbial growth. The energetic costs include the amounts of reductant (e.g. NADH or NADPH) and ATP required to support biosynthesis and thus to the carbon sources for heterotrophic growth or light required for photoautotrophic growth.

Although still at a very early stage of development, metabolic models have been obtained for cyanobacteria [e.g. *Synechococcus* PCC6803 (Knoop *et al*., 2013), *Cyanothece* ATCC 51142 (Vu *et al*., 2012)] and microalgae [e.g. *Chlamydonomas reinhardtii* (Boyle & Morgan, 2009), *Ostreococcus* spp. (Krumholz *et al*., 2012) and *Phaeodactylum tricornutum* (Fabris *et al*., 2012)]. Models are often limited to the core metabolism, which for photosynthetic organisms links light harvesting to biomass production: it includes (i) ATP and NADPH production from light reactions of photosynthesis; (ii) CO_2_ assimilation via dark reactions of photosynthesis; (iii) accumulation and mobilization of energy (carbon) storage reserves (e.g. starch, glucans and neutral lipids); (iv) nutrient (N, P, S, Fe, Mn) acquisition and assimilation; (v) generation of precursor metabolites from glycolysis, TCA cycle and nutrients; (vi) oxidative phosphorylation to produce ATP and pentose phosphate pathway to generate reducing equivalents (NADPH); and (vii) synthesis of macromolecules from precursors.

To date, most systems biology investigations of cyanobacteria and microalgae have been motivated by the potential for biotechnological applications (Wijffels *et al*., 2013), or as model systems for basic biological research (e.g. Djouani‐Tahri el *et al*., 2011; O'Neill *et al*., 2011). It is now time for oceanographers and biogeochemists to harness such models to gain a better understanding of how acquisition of resources from the environment is linked to biomass production, cell growth and population growth, along with their seasonal and other variations (Reed *et al*., 2014).

### Representing genetic constraints on adaptation (microevolution) and the integrated eco‐evolutionary response

The unconstrained response of such a trait‐based phenotype model to selection in the model environment provides a null hypothesis that natural selection produces organisms that are well adapted to their environment and that these organisms evolve to changing conditions. This defines a class of models collectively called ‘optimality‐based’ models, which provide predictions of the responses of organisms to environmental forcing based (only) on the costs and benefits of different traits or ‘behaviours’ (e.g. Talmy *et al*., 2013; Toseland *et al*., 2013; Daines *et al*., 2014).

Modelling historical evolutionary constraints on organism adaptation requires the modeller to impose some genetic constraints on the movement of organisms in a phenotypic ‘trait space’. These constraints on adaptation can be parameterized based on the results of experimental evolution studies. They require some distinction of evolutionary lineages within the model. Then, the modeller can impose lineage‐specific design constraints and macromolecular properties.

A generalization of this modelling strategy to metazoa and trophic structure can follow the same basic approach, with traits for feeding strategies and ecophysiological constraints, most fundamentally size (Kiorboe, 2010). Food web structure should then be an emergent property of the model (Loeuille & Loreau, 2005). Integration with the marine environment as represented by a GCM then provides a framework that captures key aspects of the eco‐evolutionary response (Toseland *et al*., 2013; Daines *et al*., 2014).

## Conclusions

The past decade has revealed some of the ocean's secrets through the advent of genomic‐, transcriptomic‐ and proteomic‐based approaches to describe marine microbial communities including their biogeography, metabolic potential and diversity, mechanisms of adaptation, and phylogeny and evolutionary history. The coming decade should build on that knowledge by integrating quantitative and process‐based approaches from neighbouring disciplines such as biochemistry and quantitative ecology including population genetics. Synergies arising from integrating descriptive and quantitative process‐oriented approaches will allow us to better connect genotype with phenotype and, therefore, to identify traits as a consequence of adaptive diversification. Showing that the associated phenotypes play a causal role in the ecological mechanisms driving diversification will be difficult, but is crucial for linking omics data with environmental variables and therefore integrating them in modelling biogeochemical cycles. This knowledge will enable us to link traits with environmental variables on a mechanistic basis where organisms are being modelled as the sum of their macromolecular components. Knowing their evolutionary constraints from experimental evolution studies will ultimately improve predictive capabilities of microbial responses to multiple environmental drivers such as warming and ocean acidification.
